# What Controls DNA Looping?

**DOI:** 10.3390/ijms150915090

**Published:** 2014-08-27

**Authors:** Pamela J. Perez, Nicolas Clauvelin, Michael A. Grosner, Andrew V. Colasanti, Wilma K. Olson

**Affiliations:** 1 BioMaPS Institute for Quantitative Biology, Rutgers, the State University of New Jersey, Piscataway, NJ 08854, USA; E-Mails: pperez@scarletmail.rutgers.edu (P.J.P.); clauvelin@biomaps.rutgers.edu (N.C.); michaelgrosner@gmail.com (M.A.G.); andrewco@rci.rutgers.edu (A.V.C.); 2 Wright–Rieman Laboratories, Department of Chemistry and Chemical Biology, Rutgers, the State University of New Jersey, Piscataway, NJ 08854, USA

**Keywords:** DNA looping, optimization, *J* factor, *lac* operon, Monte Carlo simulations

## Abstract

The looping of DNA provides a means of communication between sequentially distant genomic sites that operate in tandem to express, copy, and repair the information encoded in the DNA base sequence. The short loops implicated in the expression of bacterial genes suggest that molecular factors other than the naturally stiff double helix are involved in bringing the interacting sites into close spatial proximity. New computational techniques that take direct account of the three-dimensional structures and fluctuations of protein and DNA allow us to examine the likely means of enhancing such communication. Here, we describe the application of these approaches to the looping of a 92 base-pair DNA segment between the headpieces of the tetrameric *Escherichia coli* Lac repressor protein. The distortions of the double helix induced by a second protein—the nonspecific nucleoid protein HU—increase the computed likelihood of looping by several orders of magnitude over that of DNA alone. Large-scale deformations of the repressor, sequence-dependent features in the DNA loop, and deformability of the DNA operators also enhance looping, although to lesser degrees. The correspondence between the predicted looping propensities and the ease of looping derived from gene-expression and single-molecule measurements lends credence to the derived structural picture.

## 1. Introduction

The cellular environment introduces remarkable changes in DNA. The long, stiff, naturally straight, double-helical molecule appears to be much more deformable *in vivo* than *in vitro*. For example, the control of lactose metabolism in *Escherichia coli* entails the formation of DNA loops much shorter in length than those detected in aqueous salt solution. DNA tends to be highly extended in solution, with very low chances of closing into a loop having the 92 base-pair (bp) spacing found between the centers of the so-called O_3_ and O_1_ *lac* operator sequences. Formation of such a loop, against the DNA binding headpieces of the Lac repressor protein, is thought to preclude access to the genetic signals needed to transcribe proteins involved in the transport and chemical breakdown of lactose [1].

**Figure 1:**
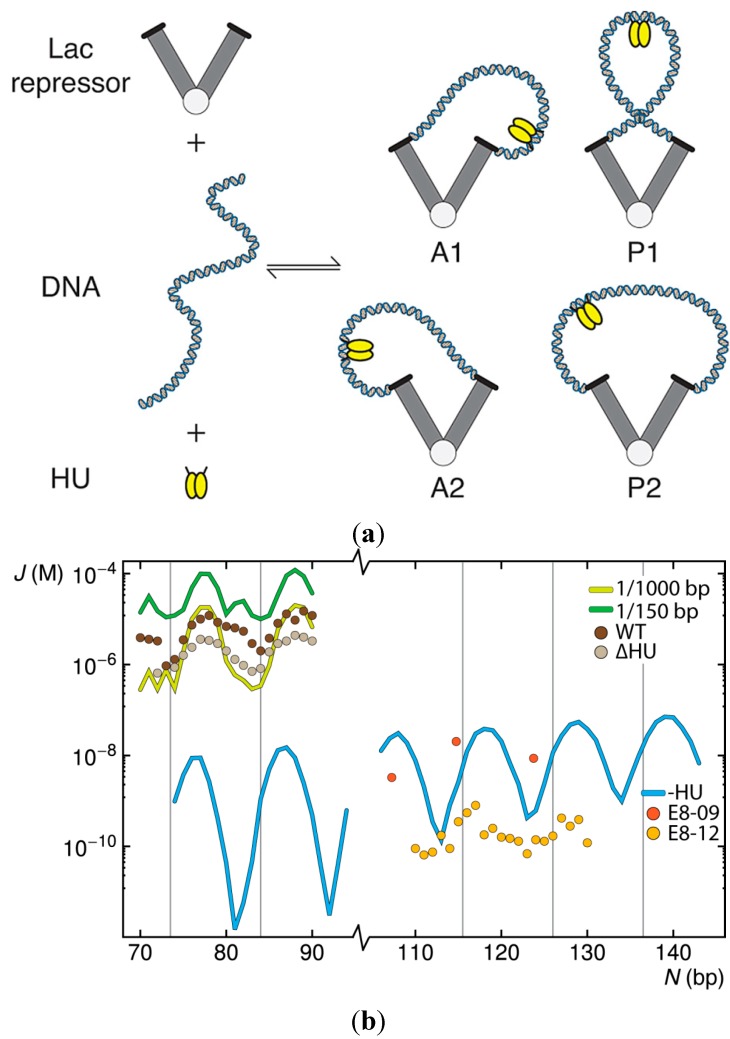
Simulated effects of HU on the ease of looping short pieces of DNA between the headpieces of a V-shaped Lac repressor protein assembly. (**a**) Schematic representation of the molecular components and resulting HU-decorated DNA loops anchored in antiparallel (A1, A2) and parallel (P1, P2) orientations [13] on the repressor; (**b**) Predicted values of the *J* factor, as a function of operator spacing, for double-helical molecules free of HU or binding one HU on average every 150 or 1000 bp compared, on the top left, with values deduced from the expression of *lac* genes in *Escherichia coli* cells containing the wild-type (WT) protein and a mutated strain (∆HU) that cannot express HU [5,14,15] and, on the lower right, with values deduced from tethered particle motion studies of DNA constructs (E8-09, E8-12) flanked at the 5'-end by a symmetric operator and the 3'-end by the natural O_1_ operator [16,17]. Computed *J* factors are connected by thin lines. Values deduced from experiments are shown by symbols. Vertical lines denote integral helical repeats of DNA.

The observed expression of the *Escherichia coli* *lac* genes thus reflects more than the mechanical properties of DNA. Key molecular components include the aforementioned repressor protein, an assembly of four identical polypeptide chains that bind specifically to the noted operators, and the naturally abundant, histone-like, architectural protein HU. The latter protein binds DNA non-specifically [2], introduces some of the largest known protein-induced deformations of the double helix [3], and stabilizes the formation of biologically functional loops as small as ~50 bp [4,5]. The Lac repressor, by contrast, binds to specific operator sites on DNA, introduces lesser distortions in the double helix [6], and interconverts between the tightly closed, V-shaped protein architecture observed in the crystalline state and an extended open form with widely separated DNA-recognition headpieces [7,8]. Moreover, the protein-bound DNA operators undergo sizable deformations with respect to the protein recognition elements, particularly at the 3'-end of the O_3_ operator [9], and the headpieces themselves may wobble with respect to the globular protein core [10]. Signals in the DNA loop may also contribute to loop formation. For example, the two hexameric promoter elements, located in the middle and near the 3'-end of the 92-bp loop, include pyrimidine-purine base-pair steps (TA:TA and CA:TG) known to be highly deformable and subject to large-scale conformational rearrangements [11,12]. The site of catabolic activator protein (CAP) binding, found at the 5'-end of the loop, also includes the same pyrimidine–purine elements. The precise orientation of the DNA operators on the protein assembly remains an open question. The operators at the ends of the loops may point toward either the inside or the outside of the repressor, yielding two classes of loops where the operators run in parallel directions and two where the operators run in antiparallel directions [13] (Figure 1a).

The treatment of protein-bound DNA molecules at the level of successive base-pair steps makes it possible to examine the contributions of HU binding, Lac repressor mobility, base-pair sequence, and operator orientation on DNA loop formation. The present article summarizes the computational approaches that we have developed to capture the structures of short DNA loops bound to the headpieces of the Lac repressor protein and presents new insights into protein-mediated DNA looping gained from these studies. The correspondence between the predicted looping propensities and the ease of looping derived from gene-expression [5,14,15] and single-molecule [16,17] measurements lends credence to the derived structural picture. Both protein and DNA play critical roles in the simulated looping. Trace amounts of randomly bound HU, deformability in the Lac repressor, and localized flexibility in the repressor-anchored DNA offer molecular rationales that help to account for the unexpectedly short DNA loops implicated in bacterial gene regulation.

## 2. Results and Discussion

### 2.1. Contributions of HU to DNA Looping

The random binding of HU has striking effects on the looping propensities of short (70–90 bp) DNA duplexes anchored to the V-shaped Lac repressor assembly. Binding of the architectural protein at the level of only one dimer per 1000 bp of DNA increases the calculated *J* factor, *i.e.*, the relative ease of looping, by two to five orders of magnitude over that predicted for ideal protein-free chains of the same length (Figure 1b). Moreover, in contrast to protein-free DNA, where the chances of looping exhibit a striking dependence on the spacing between operator sites, the *J* factors of short HU-bound loops show limited variation over the same range of chain lengths. Increasing the concentration of HU to one HU dimer per 150 bp of DNA—corresponding to the level of protein present during the exponential growth phase of *Escherichia coli*, *i.e.*, ~30,000 HU dimers [18] in the presence of a 4.6 Mbp genome [19]—dampens the amplitude of oscillations in the *J* factor with chain length and introduces secondary peaks in the looping profile. The latter peaks stem from the enhanced build-up of HU on loops of certain sizes with increase in binding levels (see below). The simulations performed at the higher concentration of HU capture the peaks and valleys in the looping propensities detected in gene-expression studies but overestimate the magnitudes of the *J* factors deduced from the experiments (points labeled WT in Figure 1b) [5,14,15]. The loops computed at the lower HU level fall within the range of observation but show greater variation with chain length than the *in vivo* data. The predicted dampening and phase shift in the chain-length dependent variation of the *J* factor upon addition of HU match trends detected in tethered-particle motion studies of longer Lac repressor-mediated loops [20]. The reported twofold enhancement in looping propensities in the latter work, however, falls short of the simulated increase in the *J* factor and the values deduced from gene-expression studies.

The computations performed in the absence of HU show both the decrease in the *J* factors derived from gene-expression studies of mutant cells that do not express HU (data labeled ∆HU in Figure 1b) and the observed oscillatory variation in loop formation with chain length [5,14,15]. The computed looping profiles, however, fall substantially below those found upon deletion of the *HU* gene, and the amplitude of the oscillations in the computed *J* factor of HU-free DNA greatly exceeds that deduced from experiment. On the other hand, the predicted difficulty in forming Lac repressor-mediated loops in the absence of HU lies within the range of values deduced from tethered particle motion studies of slightly longer loops (110–130 bp) [16,17]. The system examined *in vitro* excludes HU and any uncharacterized cellular factors that may contribute to the higher *J* factors found *in vivo*. The order-of-magnitude differences in the reported looping propensities, collected at different times with the same DNA construct (points labeled E8-09 and E8-12 in Figure 1b), are thought to reflect the different source of repressor protein in the two sets of experiments [21]. The earlier data (E8-09) coincide with the predicted curve. The later data (E8-12) show lesser variation with chain length and generally fall below the predicted *J* factors.

**Figure 2:**
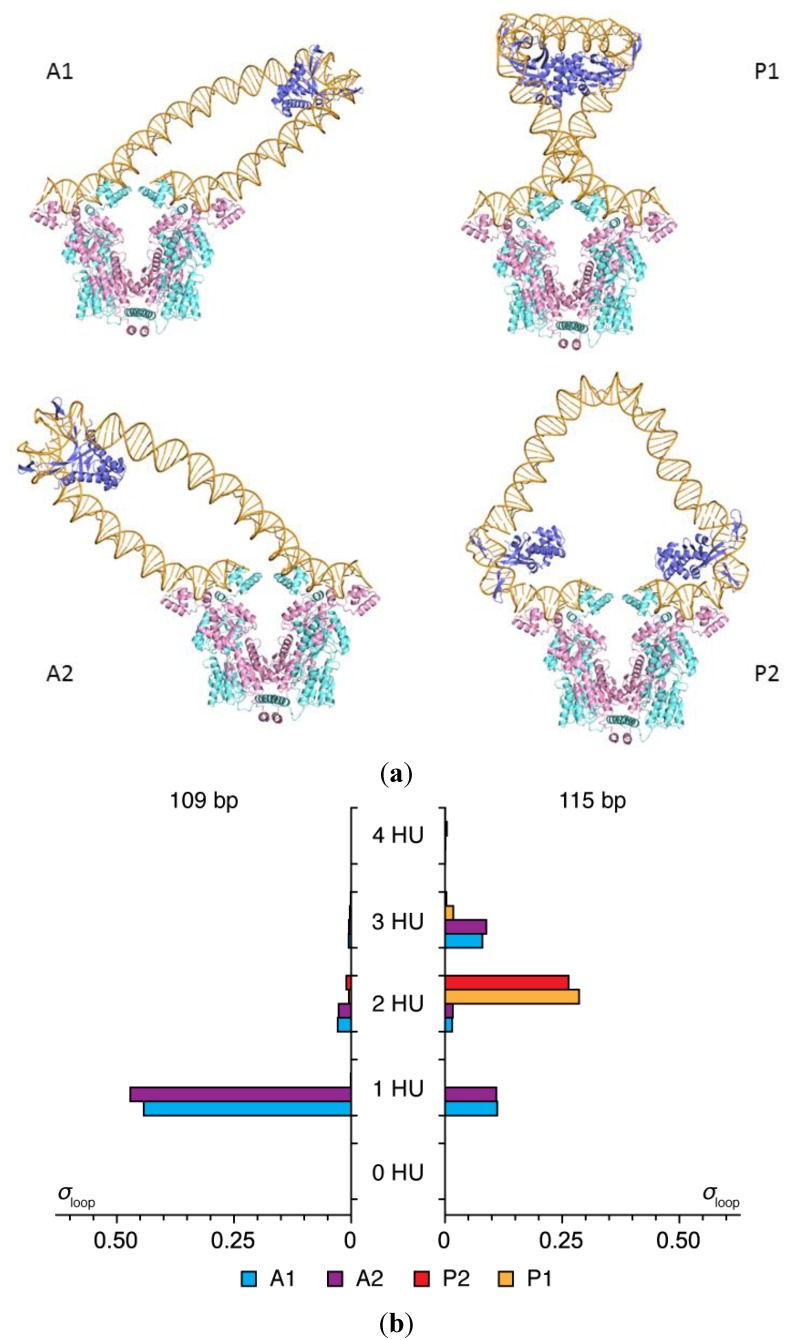
Effects of DNA chain length on the orientation of DNA and the simulated uptake of HU on loops anchored to the Lac repressor. (**a**) Molecular images illustrating the predominant configurations of DNA and the positions of HU on 109 bp (**left**) and 115 bp (**right**) loops. Images rendered in PyMOL [22] with DNA backbones are shown as gold tubes, DNA bases as gold sticks, HU chains as blue ribbons, and repressor chains as pink and cyan ribbons. Views looking down the axis perpendicular to the long axes of the globular arms of the repressor; (**b**) Frequencies of occurrence σ_loop_ of the four possible looped forms. Note the marked shift in loop orientation (A1/A2→P1/P2) and HU uptake (1→2) with the increase in operator spacing, the straight segments of DNA in the models, and the potential (highly curved) site for a third HU at the center of the P2 loop.

The HU concentration reported for the simulations refers to the probability that one HU dimer is taken up, on average, over a given length of linear unconstrained DNA (150 and 1000 bp in Figure 1b). The number of HU dimers accumulated on the DNA loops differs at low and high concentrations of protein and at different chain lengths. The loops take up more HU than the imposed binding levels at all modeled concentrations, and the uptake is greater at chain lengths where the DNA operators are out of phase with the binding sites on the repressor protein. Whereas most 109-bp loops take up a single HU dimer under binding conditions of one HU per 150 bp, the majority of HU-decorated loops of 115 bp take up two architectural proteins (Figure 2). Moreover, the HU-decorated chains anchor to the repressor in different orientations and the build-up of protein is localized. The more easily closed 109-bp chains attach to the repressor in antiparallel orientations and the less easily formed 115-bp loops in parallel orientations. The second HU, also detected in tethered-particle motion studies of less easily close loops [20], helps to align the ends of the DNA with the recognition headpieces on the repressor. The selective accumulation of protein at different chain lengths underlies the dampening of the oscillations in the computed *J* factors of HU-bound DNA compared to those of bare DNA (Figure 1b).

### 2.2. Effects of the Lac Repressor on DNA Loops

**Figure 3:**
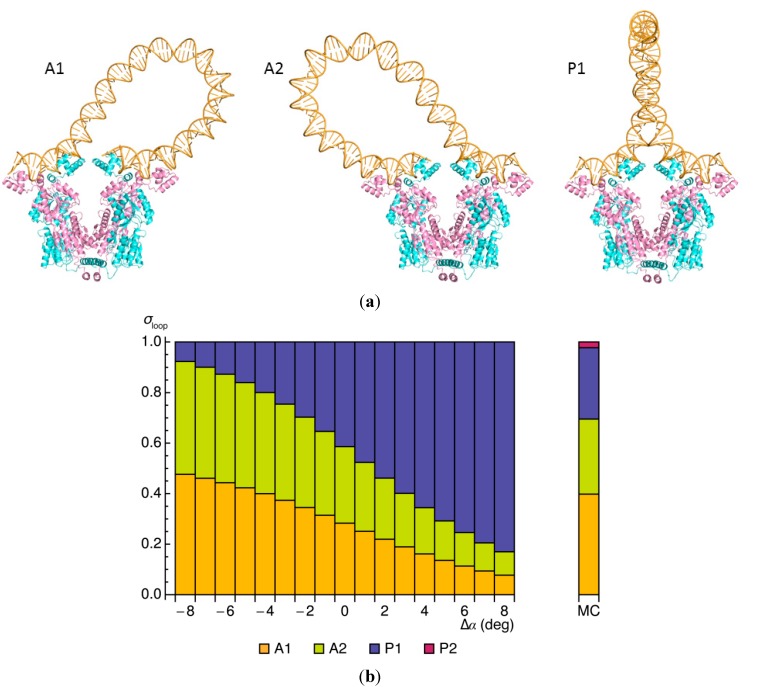
Effect of the Lac repressor on the minimum-energy configurations of 92-bp DNA loops. (**a**) Molecular images of repressor-mediated loops anchored in antiparallel and parallel orientations on the rigid V-shaped protein assembly. Note the more gradually curved pathways of the dominant types of bare DNA loops compared to the corresponding forms found in the presence of HU (Figure 2a); (**b**) Variation in the relative weights σ_loop_ of the three looped forms with small changes Δα in the angle between the protein arms, compared with the relative populations of DNA loops found to be attached to the same Lac repressor model through Monte Carlo (MC) sampling [23] (vertical bar to the right of the data obtained by energy optimization).

Surprisingly, the *J* factors found to characterize the looping of short, ideal DNA chain fragments between the headpieces of the Lac repressor exhibit a local minimum at 92 bp, the natural spacing between the O_3_ and O_1_ *lac* operators (data labeled–HU in Figure 1b). The low probability of forming such a loop reflects both the cost of bending naturally straight DNA in a closed configuration and the twisting needed to bring the terminal bases in correct register with the headpieces of the protein. The precise contributions to the energy depend upon how DNA is oriented on the protein (Table 1). For example, the bending penalty (*E*_bend_) is lower if the chain attaches to the V-shaped assembly in an antiparallel as opposed to a parallel orientation. Loops of the former type describe gradual U-turns with less straightening of DNA compared to that found in the presence of HU (Figure 2a and Figure 3a). The ease of twisting the DNA (*E*_twist_), however, offsets the cost of bending in one of the two possible parallel loops, the so-called P1 form, in which the chain adopts a more symmetric configuration with a tight turn near the midpoint of the loop. The similar elastic energies of the three kinds of bare DNA loops underlie the nearly even mix of closed forms captured in both Monte Carlo calculations and energy optimization. Occurrences of “wild-type” loops in the alternate P2 parallel form drop precipitously in the absence of HU and are therefore not discussed.

**Table 1:** Elastic energy contributions and populations of 92-bp Lac repressor-mediated DNA loops.

Loop	*E*_bend_ (*k*_B_*T*)	*E*_twist_ (*k*_B_*T*)	σ_loop_ (%)	*J*_loop_ (%)
A1	18.7	8.6	28.3	39.8
A2	18.8	8.4	30.3	29.7
P1	22.9	4.0	41.4	28.2
P2	31.4	32.9	0	2.3

σ_loop_ and *J*_loop_ are the fractional contributions of the specified loop types to the partition function and are based respectively on the exponential of the optimized energy and the *J* factor of each kind of loop.

The slight discrepancies among the populations of DNA looped forms identified with the two techniques stem, at least in part, from the difficulty in capturing configurational states that meet specific geometric criteria with Monte Carlo sampling. Examples of how slight perturbations of the imposed positions of DNA operators affect the optimized elastic energies of Lac repressor-mediated loops reveal how the approximations associated with random sampling may influence the predicted configurations of the looped structures. Here, the V-shaped protein opens or closes through small changes Δα in the virtual valence angle between the two arms (Figure 3b). Increments of ±8° limit the movements of the attached DNA to changes comparable to the range of chain displacements allowed in the Monte Carlo search—namely, deviations no more than 15 Å in the distance between terminal base pairs and fluctuations of 11.5° or less in both the global bend angle and the net twist angle. Indeed, these small perturbations in anchoring conditions have marked effects on the relative energies of loops attached in antiparallel *versus* parallel orientations on the repressor, with the former loops becoming highly favored upon slight closure of the protein and the latter upon slight opening. The changes in protein structure, however, have no noticeable effect on the overall configuration of the DNA-repressor assembly, including the contact interface thought to stabilize the interaction between the two protein arms [24].

**Figure 4:**
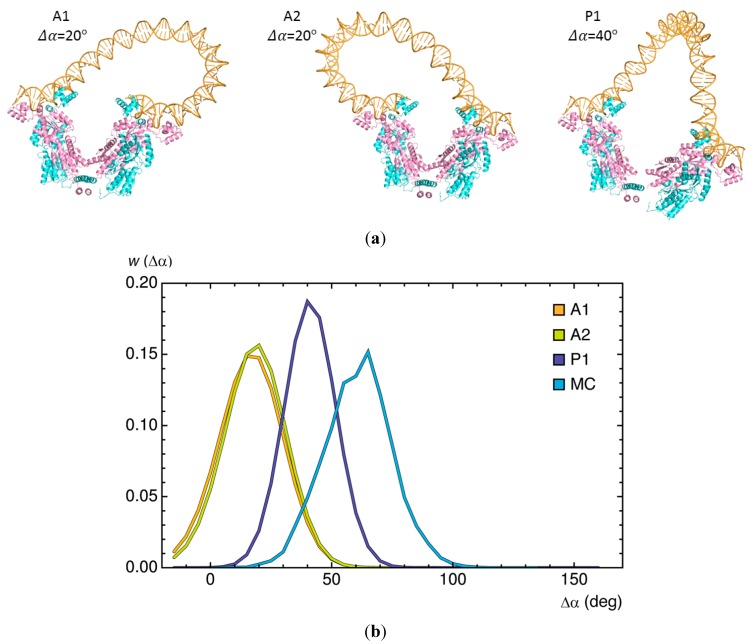
Changes in DNA looping associated with large-scale opening of the Lac repressor. (**a**) Molecular images of 92-bp loops anchored in the dominant antiparallel and parallel orientations with the change in the opening angle Δα set to values that minimize the DNA elastic energy. Note the rearrangement of DNA on opened *versus* closed repressors (Figure 3a); (**b**) Distribution of the opening angle *w* (Δα) of Lac repressor proteins anchoring DNA loops subjected to energy minimization and collected in Monte Carlo (MC) sampling. The differences in repressor opening found with the two approaches reflect the different treatments of protein, *i.e.*, precisely imposed routes of conformational change *versus* spatial forms indirectly captured in the course of DNA loop closure.

The general correspondence between the optimized energies, *i.e.*, relative statistical weights, of short DNA loops, and the probabilities of linear chain molecules satisfying the same spatial constraints allows us to consider a variety of molecular questions impractical to address with Monte Carlo methods [25]. For example, it is difficult to relate the likelihood of rare configurational events, such as the formation of 92-bp Lac repressor-mediated loops, to specific perturbations in protein structure. We next illustrate how large changes in the angle of opening Δα between the arms of the repressor affect the minimum-energy configurations of DNA loops and how loop orientation influences the degree of protein deformation (Figure 4). If fully opened, the long axes of the protein arms would lie roughly perpendicular to the axis of the connecting four-helix bundle. The constraints of DNA deformation, however, limit the degree of protein opening, with loops attached in parallel orientations more easily accommodated on the opened protein than those bound in antiparallel orientations. The changes in anchoring conditions brought about by the movement of protein lower the cost of DNA deformation, particularly the twisting contribution, in the parallel loops. A reduction in free energy comparable to the 7*k*_B_*T* difference in minimum energy found for loops attached to the opened *versus* closed protein would increase the ease of looping by roughly three orders of magnitude. By contrast, the opening of the repressor introduces a twisting penalty in loops held in antiparallel orientations and the predicted enhancement of looping is only about threefold. The 92-bp loops captured in Monte Carlo simulations bind on average to even more opened forms of the protein than predicted along the minimized energy pathway and raise the *J* factor ~7000-fold [23]. That is, the arrangements of the repressor in the latter studies include states not considered in the energy optimization.

### 2.3. Influences of Sequence and Environment on Looped Structures

**Figure 5:**
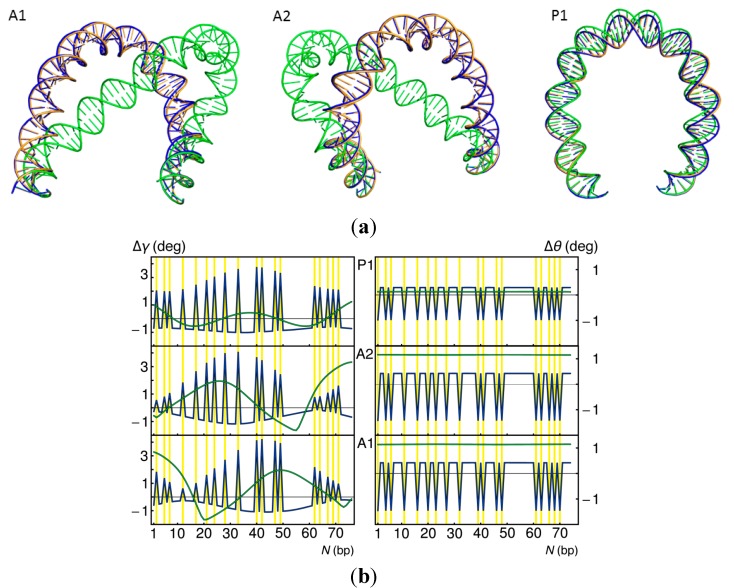
Changes in 92-bp DNA looping brought about by localized changes in the DNA model. (**a**) Superimposed images of the optimized DNA pathways found when the sites of pyrimidine-purine steps along the *lac* operon are softened (blue) and when the helix is overtwisted (green) compared to ideal DNA (gold). Terminal base pairs are constrained to the spatial positions imposed by the V-shaped Lac repressor assembly. The antiparallel loops are rotated ~45° and the parallel loop ~90° about the vertical axis relative to the images in Figure 3a; (**b**) Changes in the bending Δγ and twisting Δθ between successive base pairs along the depicted loops, with the data color-coded to match the molecular images. The yellow lines denote the locations of the pyrimidine–purine steps, where the softened DNA shows bursts of bending and untwisting. Note the relative insensitivity of the parallel loop and the responses in the antiparallel loops to imposed overtwisting.

The softening of DNA at the sites of pyrimidine–purine steps along the *lac* operon, through decrease in selected elastic constants (see Experimental Section), reduces the energy but has little effect on the overall optimized shapes of 92-bp DNA loops constrained to fit against the headpieces of the V-shaped Lac repressor assembly (blue *versus* gold DNA pathways in Figure 5a). Although the DNA bends to a greater extent at the soft steps (Δγ values in Figure 5b), the remaining residues straighten relative to those found at corresponding sites along the ideal DNA loop. The total twist of the modified chain, *i.e.*, the sum of the plotted Δθ values, also drops slightly, with the soft steps uniformly undertwisted and the remaining steps uniformly over-twisted compared to ideal DNA.

As expected for a spatially constrained DNA [26], the changes in twist along the locally softened loops are coupled to changes in bending and to the overall configurations of the loops. Individual base pairs move on average by 0.6–0.9 Å and up to 2 Å, depending on loop type, and the writhing numbers, determined along the closed pathways formed by connecting the terminal base pairs with a straight line, differ only slightly (~0.001). The computed decrease in total energy (~6*k*_B_*T* for each of the three looped states) points to an approximately 100-fold increase in the ease of looping via the assumed softening mechanism.

By contrast, the overtwisting of DNA, such as found upon the reduction of temperature [27] or the uptake of salt [28], has a dramatic effect on the configurations of certain loops (green images in Figure 5a). The increase of intrinsic twist to 36°, corresponding to a 10-bp helical repeat, displaces points along the optimized antiparallel loops by 27 Å on average and as much as 62 Å from those on the pathways of the DNA loops with an intrinsic 10.5-bp repeat. The relative displacement of base pairs is an order-of-magnitude smaller along the parallel loop, where the lesser change in shape lowers the writhing number by 0.025 (compared to reductions of ~0.25 for the A1 and A2 loops). The uniform uptake of twist along the loops (∆θ values in Figure 5b) follows the expected behavior of an ideal elastic rod [29]. An increase in energy comparable to the difference in computed minimum-energy values (~18*k*_B_*T*) would decrease the likelihood of loop formation by about eight orders of magnitude. A decrease in intrinsic twist should facilitate loop formation.

### 2.4. Effects of Operator Structure on Looping Propensities

**Figure 6:**
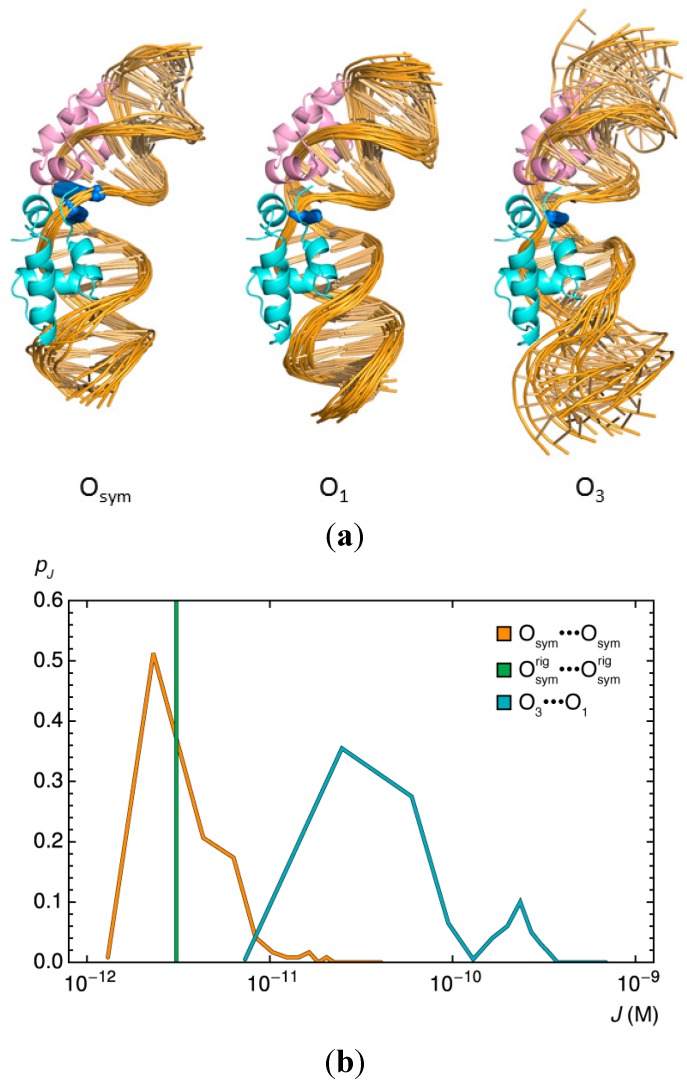
Relative ease of forming 92-bp DNA loops anchored by various Lac repressor-bound O_3_···O_1_ and O_sym_···O_sym_ operator models compared to loops terminated by identical rigid, O_sym_ operators. (**a**) Cartoon images illustrating the variability in the three operator structures. All views looking down the long axis of the kinked CG·CG step (blue) at the centerof the DNA fragments; (**b**) Distribution of the looping propensities, expressed in terms of the logarithmic values of the *J* factors, for the two classes of flexible anchoring conditions and for the rigid operator pair (
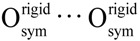
). Note the enhancement in looping with the natural operators.

The introduction on the V-shaped Lac repressor of various operator structures, representative of the spatial pathways found in NMR studies of different DNA sequences in the presence of the protein headpieces [30,31], also enhances the formation of 92-bp loops. Superposition of the NMR models in a common coordinate frame reveals distortions in DNA, with the double helix flexing to different degrees and in different directions (Figure 6a). The changes in overall bending with changes in operator sequence and the variation in apparent DNA deformability stand out in the similarly oriented molecular images. The ends of the DNA structures deduced from NMR measurements of the weak and much less curved O_3_ operator exhibit wide variations. The models of the primary O_1_ operator and the synthetic symmetric O_sym_ operator, by contrast, show limited deformations in overall structure.

The differences among the pathways of the *lac* operators have striking effects on the predicted ease of DNA loop formation (Figure 6b). The computed *J* factors of the DNA loops anchored to all 121 possible combinations of O_sym_ operator models roughly match the *J* factor, 3.1 × 10^−11^, found for the 92-bp loops formed between rigid O_sym_ operators on the same Lac repressor assembly. About half of the operator pathways enhance loop closure compared to that found with the rigid operators and about half suppress looping. The *J* factor increases more than fivefold for a few combinations of operator models and drops, in other cases, to roughly half the value found when the O_sym_ operators are rigid.

The effect of end conditions on loop formation is even more striking for 92-bp loops closed between O_3_ and O_1_ operators. The *J* factors of loops anchored to any of the 200 O_3_···O_1_ model combinations exceed the value found with rigid O_sym_ operators (Figure 6b). Moreover, the looping enhancement is 20–30 times larger than that from the latter simulations for DNA fragments attached to about two thirds of the operator models and nearly two orders of magnitude larger for loops formed with a quarter of the models. The predicted enhancement of looping is tied to specific operator pathways, notably the straightened O_3_ pathways where DNA peels away from the repressor [9].

## 3. Experimental Section

### 3.1. Protein-Decorated DNA Model

This work takes advantage of methods that we have developed to study the properties of spatially constrained DNA molecules, including Lac repressor-mediated DNA loops. Models of DNA are constructed, one base-pair step at a time, from sets of rigid-body parameters that specify the three-dimensional arrangements of successive base pairs [32,33]. Protein-free segments are subject to a potential that allows for elastic deformations of the long, thin molecule from the canonical B-DNA structure [34]. The base-pair steps are assigned the elastic properties of an ideal, inextensible, naturally straight DNA helix, with bending deformations (±4.84°) corresponding to a persistence length of nearly 500 Å, fluctuations in twist (±4.09°) compatible with the topological properties of DNA minicircles (that is, twisting 1.4 times more restricted than bending) [35,36], and a helical rest state with 10.5 bp per turn. The enhanced deformability of the pyrimidine–purine steps introduced in selected loops is described by a model that allows for angular fluctuations ~1.5 times those available to the other base-pair steps, *i.e.*, root-mean-square deviations in the bending and twisting components ~1.5 times those assigned to the canonical helix.

The DNA attached to the Lac repressor is defined in terms of the rigid-body parameters of the base pairs found in known operator-bound structures (described below). The HU-bound fragments are assigned the sets of parameters that describe the pathways of DNA adopted in the four crystal complexes with the *Anabaena* protein [3] and are introduced at random along the looped DNA [23]. The probability that a site is occupied depends upon the concentration of free HU, its DNA association constant, and the concentration of DNA base pairs. The DNA is examined, one base pair at a time, in random order, and a decision is made whether or not to place the protein. If the HU is placed, the 14 base-pair steps that make up the binding site are assigned the step parameters in one of the reported crystal structures and the protein is incorporated as a rigid “side group” of the DNA [37].

The Lac repressor is subjected to large-scale motions that preserve its globular elements and follow directions consistent with experimental observations [7,8]. The opening of the repressor to an extended state is affected via a rotation of one of the two large globular arms of the tetrameric assembly about a “bending” axis perpendicular to the long axes of the two arms and passing through the point of closest approach between those axes (Figure 3 and Figure 4). The disordered state of the polypeptide backbone in this region of the structure lends support to the idea that the protein may deform by such a mechanism. The reference state is a V-shaped model of the full protein–DNA assembly constructed from known crystallographic components [9,24], e.g., superposition of the protein atoms in one of the dimeric DNA-bound repressor structures [38,39] on the corresponding atoms in the tetrameric form of the protein without DNA-binding headpieces [6]. Here, for simplicity, the opening of the repressor is assumed to be “free”, with no energy penalty associated with the disruption of the small contact interface believed to stabilize the V-shaped form [24]. Introduction of a penalty term proportional to the surface area of the contact interface in the closed complex does not change the general findings.

The subtleties in structure associated with the binding of different DNA sequences are treated by superimposing the solution structures of the Lac repressor headpiece bound to different operators [30,31] on V-shaped models of the full protein–DNA assembly [9,24]. Given the uncertainty in the orientation of the headpieces on the tetramer, the complexes are placed in two directions on each arm of the V-shaped models, with the 5'–3' direction of the operator pointing toward either the inside or the outside of the full structure. The GC base pairs at the centers of the natural (O_1_ and O_3_) operator-headpiece complexes [31] overlap the corresponding base pair in the O_1_-containing crystal structure [39], and the highly kinked CG base-pair steps at the centers of the symmetric (O_sym_) operator complexes [30] coincide with the corresponding step in the O_sym_ crystal complex [38]. The perturbations in these systems affect the positions and orientations of the DNA at the two ends of the tethered loops and the ease of accommodating an intervening length of DNA. The rigid DNA operators introduced in other computations follow the pathway taken by the symmetric operator in the crystalline state [38].

### 3.2. Looping Simulations

The ends of the protein-bound operator fragments are used as anchoring points in Monte Carlo simulations of the likelihood of DNA loop formation and in the optimization of energetically preferred spatial pathways. As noted above, in the absence of knowledge of the directions in which the operators attach to the arms of the repressor, each operator is placed in two orientations on the protein-binding headpieces and loops of four different types are generated for a given repressor–operator model.

The ease of DNA loop formation is estimated from the fraction of simulated configurations of a linear molecule with terminal base pairs positioned so as to overlap the base pairs at the ends of the repressor–operator assembly. The formation of a successfully closed loop is detected by adding a phantom base pair to the 3'-end of the DNA and checking its coincidence with the first base pair of the loop [23,34]. The joining step incorporates the rigid-body parameters, found in the complex of repressor with operator DNA, that relate the coordinate frames on the anchored base pairs. The latter parameters depend upon the precise structure of the full repressor–operator assembly and thus vary as the protein opens or the DNA operators fluctuate about their rest states. The probability of DNA looping is reported in terms of the Jacobson–Stockmayer *J* factor, the well-known ratio of the equilibrium constant for polymer ring closure compared to the bimolecular association of a linear molecule of the same length and composition [40]. The greater the value of *J* is, the lower the free energy of DNA is and the greater the likelihood of looping is.

Representative configurations of DNA chains are obtained, as described previously [34], by direct Monte Carlo enumeration using a standard Gaussian random-number generator [41] and a modification of the Alexandrowicz half-chain pairwise-combination technique [42]. The random placement of HU on DNA includes corrections for the potential overlap of bound proteins and the generation of partial binding sites on simulated half chains as detailed in full elsewhere [37]. The configurations of DNA chains capable of looping between the headpieces of the repressor are identified from the spatial disposition of terminal base pairs. The six step parameters that relate the first and the last (phantom) base pairs should be null in a perfectly closed loop, and the end-to-end vector **r**, the global bend angle Γ, and the net twist angle ω should also be zero. Because the chances are very low that the added base pair will superimpose perfectly on the first base pair in any simulated structure, the end conditions are relaxed and only configurations that fall within the following bounds are classified as looped: (i) |**r**| ≤ 15 Å; (ii) cos Γ ≤ 0.98; (iii) cos ω ≤ 0.98. Samples of 10^17^ random chains, collected in 5–8 h on a high performance computer cluster for a given combination of loop anchoring conditions, typically yield ~500 possible arrangements of less easily formed loops (*J* factors of ~10^−14^) and over 500,000 examples of more readily closed structures (*J* factors of ~10^−11^).

The effects of specific changes in the protein-DNA model on relative looping propensities are also estimated from the relative statistical weights of the minimum-energy structure that meets the desired conditions compared to the reference state, *i.e.*, e^−∆*E*^, where ∆*E* = *E_i_* − *E*_0_ is the difference in energy between the modified state and the ideal isotropic DNA loop anchored to the V-shaped repressor.

### 3.3. Loop Optimization

Minimum-energy looped structures are obtained with a new procedure that makes it possible to optimize the potential energy of elastic deformation of a collection of base pairs, in which the positions and orientations of the first and last pairs are held fixed [43]. Rather than describe the chain in terms of the six standard rigid-body parameters, we introduce a new set of variables that keep track of the vectorial displacements of successive base pairs in a global reference frame. The choice of variables makes it possible to take the imposed spatial constraints into direct account and to use unconstrained numerical optimization methods. In other words, a constrained optimization problem is transformed into an unconstrained one, and the numerical implementation is simpler and more robust. By contrast, the standard rigid-body parameters associated with a DNA base-pair step are expressed in a dimeric frame midway between the reference frames of adjacent base pairs so that the magnitudes of the local translational components are independent of chain direction [44,45].

We consider a collection of base pairs with imposed positions on the first and last residues. That is, we specify the three components of the end-to-end vector and the three rotational degrees of freedom that describe the relative displacement and orientation of coordinate frames on the first and last base pairs. We replace the set of base-pair step parameters with the equivalent new set of independent variables, termed the step degrees of freedom. The dependence of these degrees of freedom on the base-pair step parameters can be obtained analytically. In addition, the imposed end-to-end vector and end-to-end rotational constraints can be expressed such that the step degrees of freedom for the last base-pair step are functions of the degrees of freedom of all the other steps. In other words, we reduce the dimensionality of the problem by taking into account the boundary conditions (the dimension is reduced from 6(*n* − 1) to 6(*n* − 2), where *n* is the number of base pairs). The gradient of the potential energy of elastic deformation for the collection of base pairs and the Hessian matrix are also obtained analytically. The minimization procedure is implemented as a gradient-based optimization, e.g., conjugate gradient or BFGS (Broyden–Fletcher–Goldfarb–Shanno) optimization, in the reduced space of the step degrees of freedom. Full details are presented elsewhere [43].

### 3.4. Loop Characterization and Manipulation

We describe the local structures of the DNA loops in terms of the bending and twisting of the constituent base pairs. The degree of bending is taken as the angle γ between the normals of successive base pairs, and the twist angle θ is based on a discrete ribbon constructed from the origins and reference frames of successive base pairs [46]. In contrast to the twist angle included in the six rigid-body parameters describing the spatial arrangements of successive base pairs [44], the twist reported here, the so-called twist of supercoiling, can be combined with the writhing number of a closed structure to obtain the correct linking number [46,47]. The reported loop lengths *N* correspond to the number of base-pair steps between the centers of bound DNA operators, *i.e.*, seven of the 14 steps attached to each arm of the modeled repressor–operator assembly plus the DNA steps subjected to configurational changes (78 such steps for the 92-bp *lac* loop). We also take advantage of a feature in the 3DNA software that allows a user to look at multiple structures from a common perspective [48].

## 4. Conclusions

How the naturally stiff DNA double helix forms the short loops implicated in the regulation of bacterial genes has long been a mystery. New computational approaches that take direct account of the known structures and fluctuations of protein and DNA are making it possible to envision ways in which the loops might assemble and function. For example, trace amounts of the nonspecific architectural protein HU enhance the computed likelihood of loop formation several orders of magnitude beyond that predicted for protein-free DNA, to levels comparable in value to those deduced from the expression of *lac* genes in *Escherichia coli*. The computed ease of looping DNA between the headpieces of the Lac repressor protein also increases, albeit to a lesser extent, if the tetrameric protein opens from the V-shaped arrangement found in the crystalline state, if parts of the DNA loop soften or unwind, or if the DNA operator fluctuates among the ensemble of structures determined from NMR measurements.

The nearly one-to-one relationship between the relative statistical weights of 92-bp Lac repressor-mediated DNA loops identified through Monte Carlo sampling and energy minimization now makes it possible to address a broader range of systems and problems. We can thus perform rapid optimizations to probe rare looping events or to examine specific contributions of protein and DNA in certain kinds of loops. Here, we showed how a particular type of repressor opening enhances the likelihood of forming 92-bp Lac repressor-mediated loops and how simple modifications of local chain deformability and intrinsic structure change the structures and relative energies of looped DNA. We are in the process of examining how other types of repressor motions contribute to loop formation and how more realistic treatments of DNA fine structure and local deformability, such as anistropic bending or intrinsic curvature, influence the predicted results. We can also take explicit account of the supercoiling of DNA essential for Lac repressor-mediated DNA looping and can introduce loops within larger molecular contexts, such as topologically isolated domains within a long plasmid.

Earlier treatments of the *in vivo* looping properties of DNA [49,50] were phenomenological in the sense that the effects of the molecular system on DNA chain closure were subsumed in the parameters of simple models fitted to the data. For example, DNA treated purely as an ideal elastic rod appears to soften in the presence of HU as gauged by the force constants needed to account for the observed dependence of the *J* factor upon chain length. By contrast, the present work takes direct account of the structures and fluctuations of protein and DNA and makes it possible to decipher the precise contribution of each component to loop closure. The incorporation of HU allows DNA to adopt spatial pathways normally inaccessible to the stiff, naturally straight duplex, and the propensity of DNA to remain straight restricts the sites of HU-induced deformations. That is, even though HU binds in a sequence neutral fashion to unconstrained linear DNA, the architectural protein must accumulate at specific sites in order to bring the ends of the looped fragment in register with the repressor headpieces. Thus, the DNA stiffens and the HU binds specifically in the sense that the duplex is extended and the HU is localized at particular sites. The predicted rearrangements of DNA in the presence of HU, including the altered spatial disposition of regulatory elements and the potential effects of topologically localized proteins on gene expression, introduce a new perspective on protein-mediated DNA looping worthy of further investigation. In this regard, we are working on improvements to the potential functions that will allow us to include electrostatic interactions of DNA and protein and estimates of the cost of protein deformation in future studies.
